# Controlling your contents with the breath: Interactive breath interface for VR, games, and animations

**DOI:** 10.1371/journal.pone.0241498

**Published:** 2020-10-29

**Authors:** Jong-Hyun Kim, Jung Lee

**Affiliations:** 1 School of Software Application, Kangnam University, Yongin, Gyeonggi, Republic of Korea; 2 School of Software, Hallym University, Chuncheon, Gangwon, Republic of Korea; Politechnika Slaska, POLAND

## Abstract

In this paper, we propose a new interface to control VR(Virtual reality) contents, games, and animations in real-time using the user’s breath and the acceleration sensor of a mobile device. Although interaction techniques are very important in VR and physically-based animations, UI(User interface) methods using different types of devices or controllers have not been covered. Most of the proposed interaction techniques have focused on screen touch and motion recognition. The direction of the breath is calculated using the position and angle between the user and the mobile device, and the control position to handle the contents is determined using the acceleration sensor built into the mobile device. Finally, to remove the noise contained in the input breath, the magnitude of the wind is filtered using a kernel modeling a pattern similar to the actual breath. To demonstrate the superiority of this study, we produced real-time interaction results by applying the breath as an external force of VR contents, games, and animations.

## Introduction

Recently, as the VR and AR(Augmented reality) fields have emerged as hot issues, related industries, such as game, medical, military, simulation, and film, are developing very much. Accordingly, various UI methods are required in the market [1–3]. In addition, with the introduction of low-cost VR HMDs(Head mounted displays) such as Oculus Go, not only games but other types of VR contents are spreading to the general public. Recently, researches have been conducted to capture the movement of a finger using optical markers to realistically express the interaction [4].

Various interface techniques and devices related to VR have been proposed, but most of them focus on gesture recognition using hands and feet [1–3]. User interaction technology is greatly affecting not only VR/AR but also mobile games and medical markets. However, most mobile applications are controlled only by user interaction based on screen touch, and in VR, contents are controlled based on hand motion. Although the UI using hands and feet can intuitively control graphic contents, it is difficult to express the movement of an object other than a person. For example, the interaction of objects using wind and the diffusion of paint are difficult to express with touch-based interfaces. In addition, users with inconvenient hands and feet cannot use these contents. This lack of interface diversity also affects the development of the content market.

We often see interface methods that utilize tools like brushes, but this is just one type of interface based on hand motion. In this study, the user directly blows the breath on the mobile device to determine the direction and magnitude of the wind and controls the contents by calculating the control position to which the breath is applied using the acceleration sensor. Based on this, we propose a new UI framework that performs interaction in real time in VR, game and animation. In this paper, we produced interactive results in which the user controls the physically based fluid simulations, VR contents, and games by breath in real time according to the position and orientation of the mobile device and the magnitude of the breath. The contributions of this study are as follows.

1. We propose a method to control the direction of the breath according to the angle between the user’s point of view and the mobile device. Also, even when the mobile device suddenly rotates, the rotation value can be stably calculated using the angle-based blending technique.
2. We present an interface technique that changes the control position according to the orientation of the mobile device. Based on this technique, the user can easily change the target position to which the breath is applied, rather than controlling the contents at a fixed position.
3. In order to remove noise included in the input breath, we propose a filtering method through a kernel similar to the actual breath.
4. The control points and breath orientation and magnitude calculated by the mobile device were applied to the VR, games, and animation environments for real-time control.

### Related work

Input methods that can be used for interaction of mobile devices include camera and gyroscope sensor. Video mouse is an input method developed similarly to a mouse using the RGB image of a camera. This technique obtains two-dimensional coordinate values by moving on a pad on which a specific pattern is drawn. Another interaction approach is to use a gyroscope sensor to navigate, pan, or scroll up, down, left, or right. These methods enable basic operations such as navigation, panning, and scrolling on the screen by tilting the mobile device up or down or left and right [5].

In VR content, the sensory experience of how realistically the user acts and reacts to it is important. Recently, with the development of various sensors and information processing technology, it is possible to accurately recognize the motion of joints, hands and feet, and the development of HMD devices gives vivid visual immersion as if in a real environment. This makes natural and intuitive visualization and interaction possible in the VR environment. Recently, in order to maximize the reality of VR, active researches are being conducted not only in the display field but also in the auditory field that realizes a sense of space and the haptic field that implements tactile sense [6–9]. Kinect, Leap motion, Myo, etc. are well known devices that can recognize a user’s gesture. Kinect provides a depth map with RGB images, so it can be applied to more diverse fields. In particular, this device is effective for tracking the joints of the body and can track several people at the same time [10]. Leap motion is a device specialized for recognizing hand position, finger joints and hand gestures and shows very small tracking errors [1]. The Myo gesture control armband is worn on the user’s arm and measures the EMG signal to recognize movements such as shaking hands, moving fingers, and rotating the arm [11, 12].

There are studies in the field of VR that suggest handheld grabbing tools. Kitamura et al. attempted to catch virtual objects using virtual chopsticks [13]. This method uses motion mapping to catch objects by analyzing the finger motion patterns that appear when the user chops them. In addition, Daichi’s artworking [14], MAI painting brush [15], and ToolDevices [16] designed VR devices in a form similar to real tools by implementing virtual tools and controllers to enable real-world physical representation, and further developed the concept of VR tools. Recently, Yang et al. has proposed a virtual tool for grabbing virtual objects using patterns similar to real chopsticks [17].

Katzakis et al. designed a controller that can manipulate 3D objects using a smartphone by proposing a plane-casting technique using distance [18]. This method stably manipulated the 3D object within a certain distance and determined the movement and direction of the 3D object according to the hand touch and the direction of hand movement. In plane-casting method, the shape of the tactile sensor (not necessarily a touch-display) is cast inside the scene at some distance from the user’s viewport. In addition, FERC(Fixed-point extended ray-casting) method for performing ray-casting at a fixed position, and PaM(Point-and-move) method for moving a smartphone while the user pushes a button in a direction to move a 3D object. These techniques control the contents taking into account the touch patterns of the fingers [19].

Mobile devices are equipped with high-quality cameras, and they are widespread in our daily lives. As a result, mobile-based AR-related apps have made significant advances in recent years. Most AR apps running on mobile devices can experience content while walking around [20, 21]. However, walking around the streets while enjoying AR content is at risk of various kinds of accidents due to the limitations of multitask processing, and methods for preventing such accidents are proposed. These techniques often employ a variety of devices, such as ultrasonic waves and infrared sensors, to recognize the surrounding environment.Kang et al. presented an obstacle detection system for pedestrians using mobile AR applications [20]. The system analyzes the images from the camera to extract the feature points and determines whether the feature points come from an obstacle ahead of the path and detects the presence of an obstacle. Shin et al. developed a wearable system that uses ultrasonic sensors to detect nearby obstacles and calculate the direction to avoid them [22]. In addition, there is a system that displays obstacles around the user by connecting a depth camera to the mobile device [23].

Many VR studies improve the immersion by providing users with very realistic VR environments, objects and interactions through senses such as vision, hearing and tactile sensations. VR devices such as HMD, data gloves, and motion capture provide users with realistic visual and tactile experiences through interaction with objects. Based on these experiences, various VR applications have been developed, and studies have been focused on developing interaction techniques that can be efficiently, intuitively and conveniently manipulated by users. There have been techniques for physical interface and interaction that enable easy control of motion and expression in virtual environments using gaze and gestures [24–26]. Recently, interaction in PC environment is supported at low cost through controller of Oculus Touch and HTC Vive [27–29], and in mobile VR environment, a method of manipulating virtual objects using gaze and hands based on gyro-sensors of smart phones is proposed [30–32]. In addition, a study has been proposed that can analyze behavior, gestures, and movements by capturing hands through markers to directly reflect user behavior in virtual environments [4]. There is also an interaction technique related to user walking in a VR environment [33, 34], and recently, a motion platform that can express free movement by recognizing walking has been proposed [35]. In this interaction process, it is important not only to express the user’s behavior, but also to accurately measure the user’s force and feedback the physical reaction to the virtual environment. A haptic system that expresses the physical interaction based on the user’s force, such as the haptic interface proposed by Jayasiri et al., has been studied to improve the user’s presence in immersive VR [36].

In addition, there is also a study on VR application that is used as a VR therapy system [37], which has been expanded to be used for the treatment of phobias or depression. Away from a system that unilaterally asks a question or shows information, a VERT (Virtual Reality Exposure Therapy) system that utilizes user’s physiological signals has been proposed. Although not a VR or game, there are systems that process speaker recognition using breath biometrics [38]. They define breath data as a human unique fingerprint, and use it for speaker recognition to show speaker recognition results with high accuracy compared to existing classification algorithms. The use of breath data in this technique is similar to the method proposed in this paper, but it is different from our method in using the existing template matching technique-based classification technique to calculate breath features. In this study, we propose a method to control physically-based simulation and VR contents using a breath interface. We calculate the breath magnitude by filtering the breath data received from the sound sensor, determine the wind direction using the angle calculated from the mobile device and the user’s viewport, and efficiently control the contents through these two features.

Ultra [39] and Infraasee [40] are equipped with ultrasonic sensors and infrared sensors on mobile devices to detect ground changes such as stairs or subway station platforms. However, these sensors are usually not included in mobile devices, and there is an additional cost to install them. To solve this problem, new interface technologies using only embedded sensors have been proposed. WalkSafe analyzes images from the mobile device’s rear camera with machine learning to detect vehicles approaching users [41]. Zhou and Zhengjuan analyze data obtained from sensors embedded in mobile devices such as acceleration sensors and gyroscopes to determine whether a user is walking [42]. Tang et al. suggested a way to detect the tactile paver in a given camera image to distinguish dangerous roads from safe sidewalks [43]. Most interface technologies developed based on mobile devices process or analyze images obtained from cameras, and acceleration and gyroscope sensors are mainly used to obtain direction information. This paper also proposes a new interface technology that determines the intensity and direction of the breath and controls various contents from the sound received by the microphone sensor embedded in the mobile device.

## Proposed framework

This section describes each step of our method (This research is approved by the ethics committe in school of software application at Kangnam University).

### Calculating breath with angle blending

**Fig 1:**
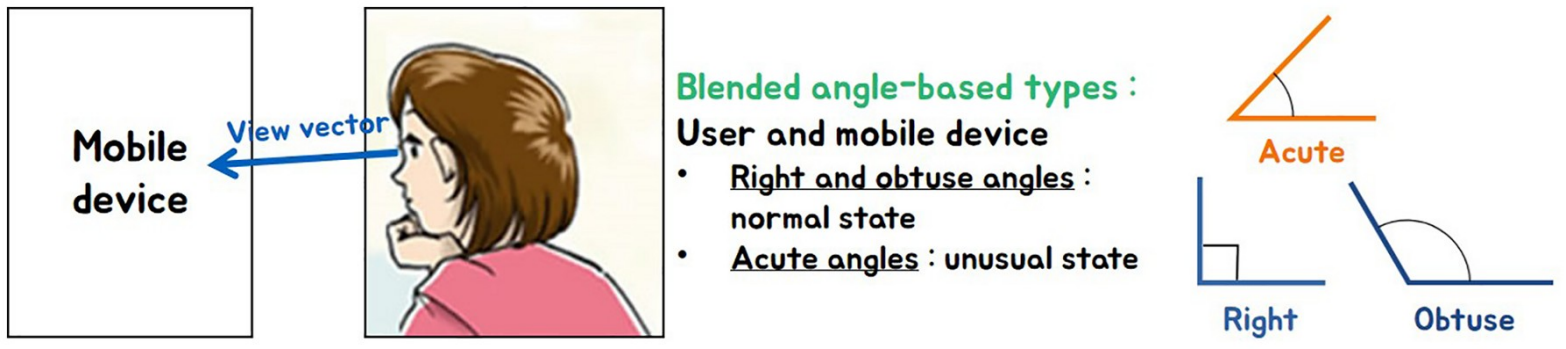
Two states based on the angle between mobile device and view vector.

In this study, the direction of the breath is determined by calculating the angle between the mobile device and the user’s viewpoint (see Fig 1). The user’s point of view is calculated using an approximation based on angle, not the exact position the user looks at the mobile device. Based on the angle calculated based on the direction of the user’s viewing of the mobile device, the right or obtuse angle is defined as the normal state, and the acute angle is defined as the unusual state.

**Fig 2:**
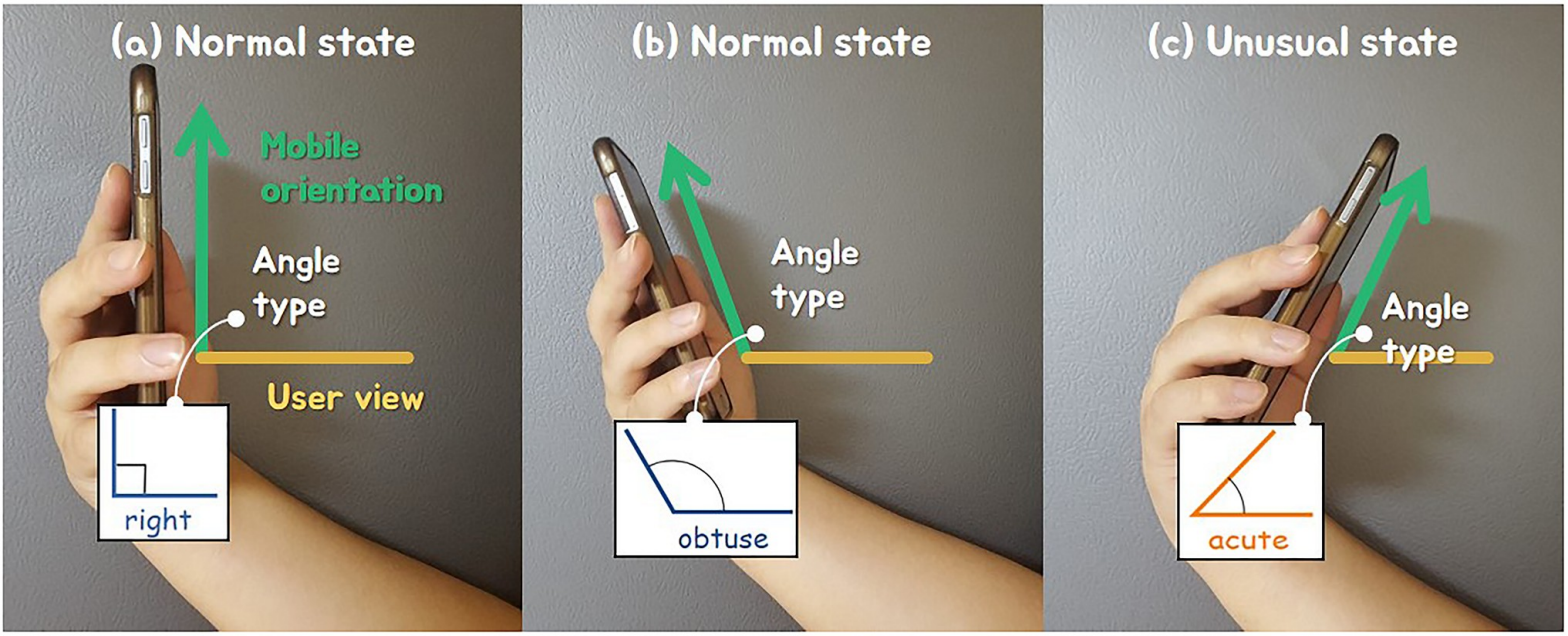
Classification of wind direction according to three angle types.

To calculate the angle at which the mobile device is held, we need to calculate the angle between the tilted orientation of the device and the front direction of the user. In this paper, we can know the orientation and movement of the smartphone in 3D space by using its acceleration sensor. The default state is when the angle between the user’s viewpoint and the *Y*-axis of the acceleration sensor is at right angles. In this state, the wind direction is set upward (see Fig 2a). In most cases, the user holds the mobile device at a right angle or an obtuse angle. In both types, the wind direction is set upward (see Fig 2b). On the contrary, when the mobile device is held at an acute angle, the wind direction is set downward (see Fig 2c).

**Fig 3:**
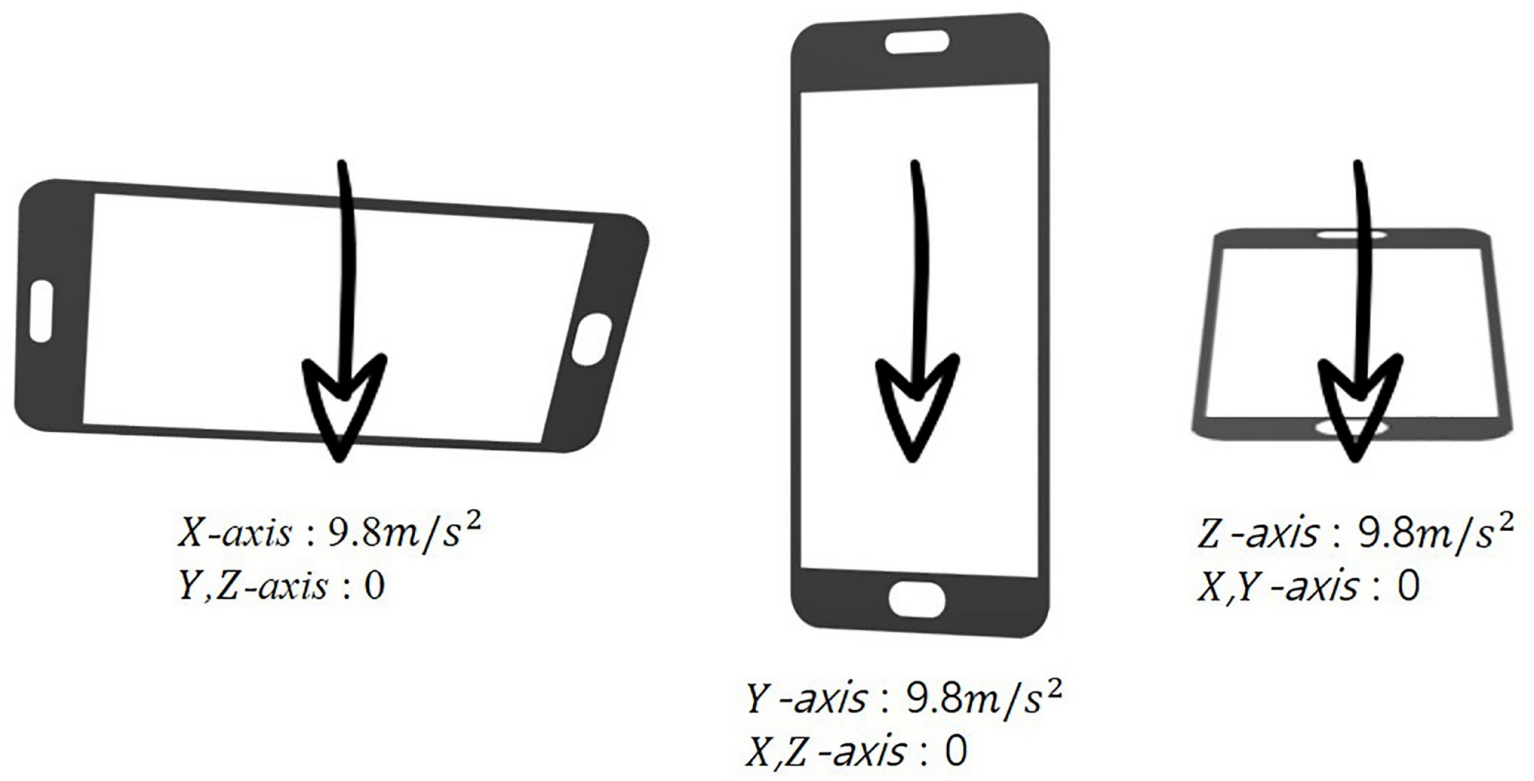
Influence of gravity on mobile device condition.

Since the acceleration sensor is always affected by gravity, the measured acceleration value is 9.8 *m*/*s*^2^ if the mobile device is stationary (see Fig 3). Therefore, the orientation of the mobile device is calculated using the vector of acceleration measured at each axis, assuming the device is stationary (see Eq 1). We put the weight *α* to increase the magnitude of the direction vector so that the wind gets stronger whenever the angle increases from right to obtuse (see Eq 1 and Fig 5).

$$\begin{array}{c} tan(\theta )=\frac{x}{z},∴\theta =atan2(\frac{x}{z})\frac{180}{\pi }\alpha , \end{array}$$ (1)

**Fig 4:**
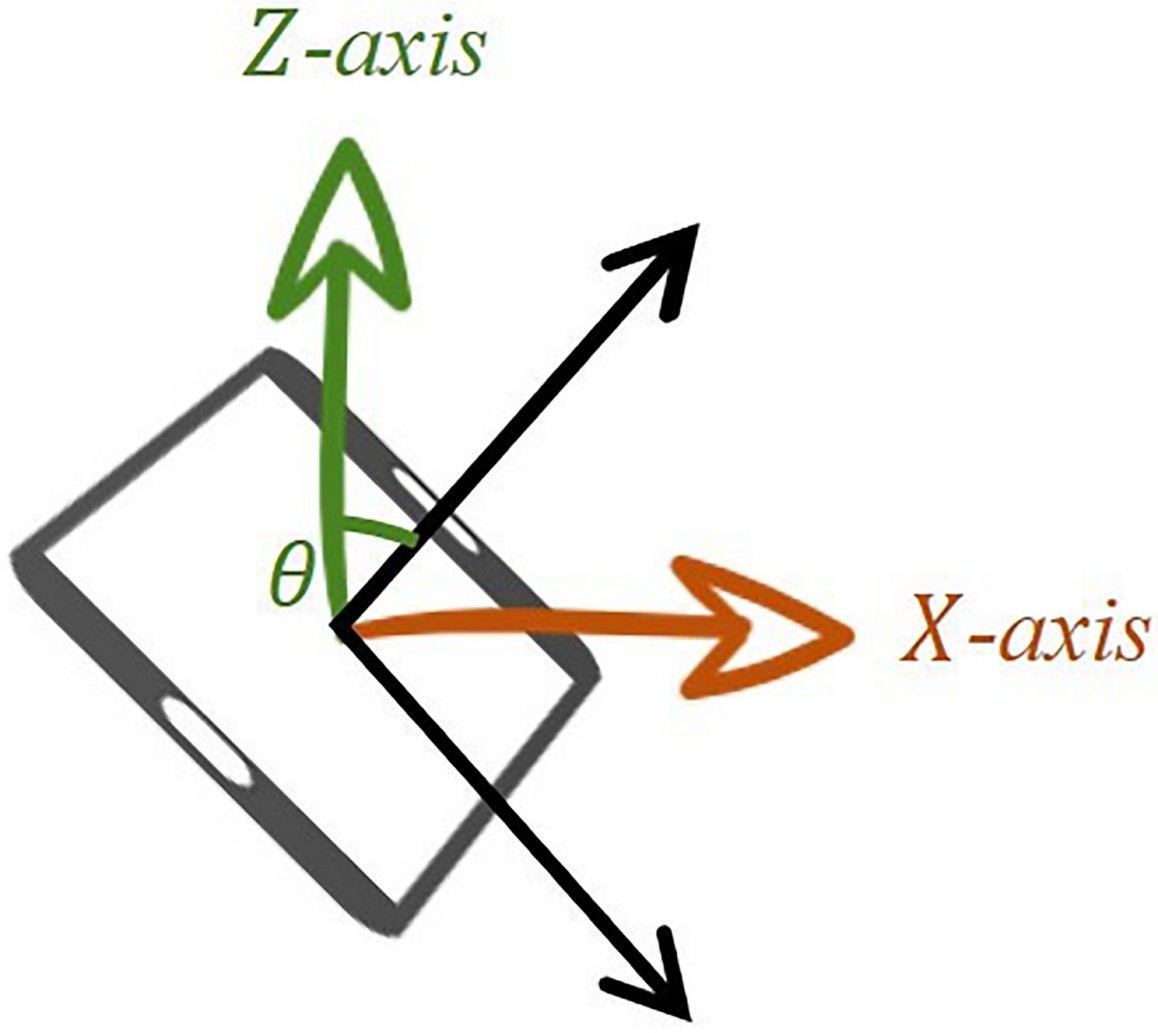
Example.

**Fig 5:**
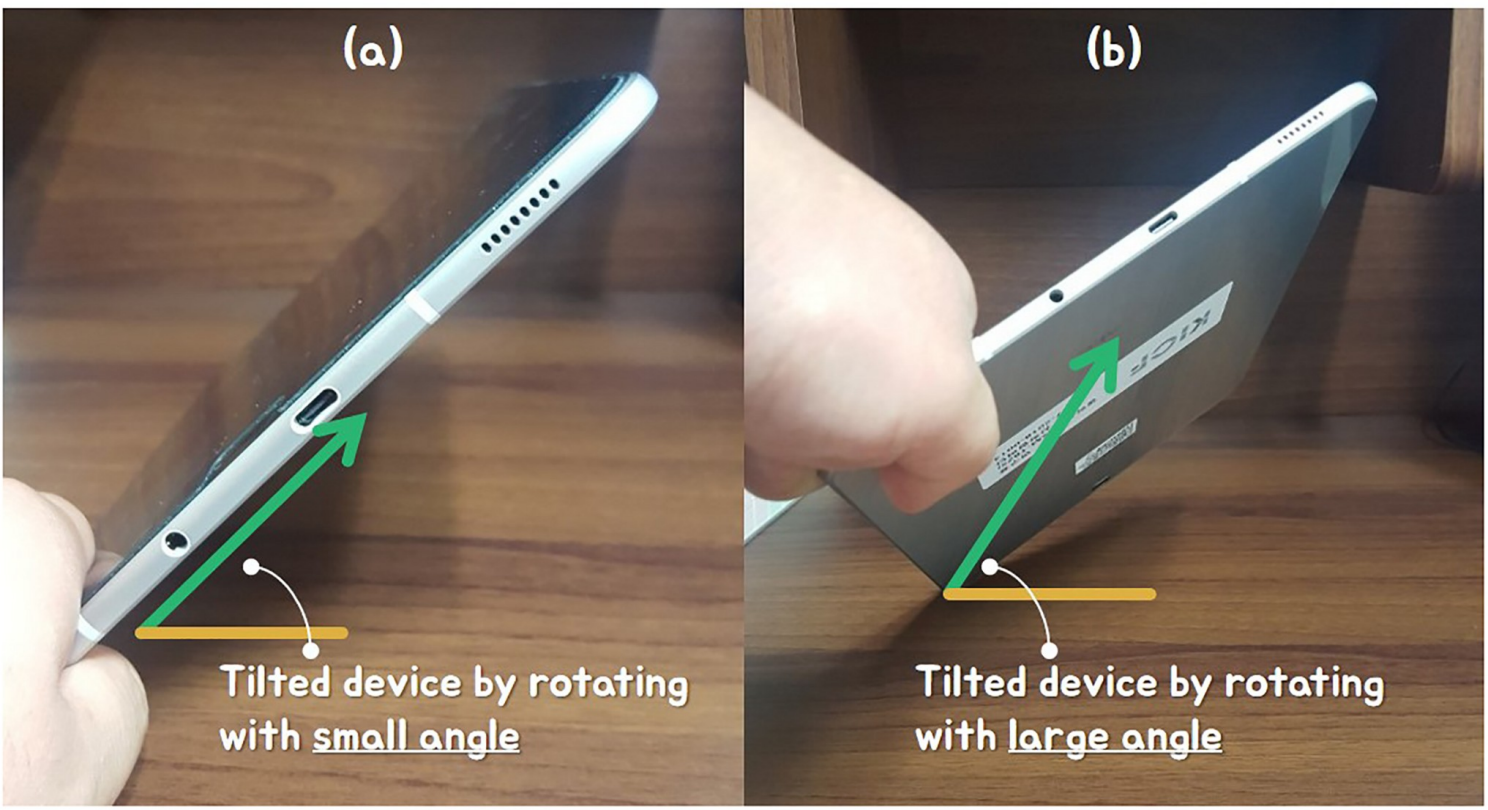
Various rotation angle on mobile device.

The figure above shows the results of assuming that the sensor moved as much as theta between the *X*-axis and the *Z*-axis (see Fig 4). In Eq 1, because atan2 returns the radian value, it is multiplied by $\frac{180}{\pi }$ and changed to degree units. As a result, the mobile device is defined as the normal state when the mobile device forms a right angle or obtuse angle with the user’s line of sight, and is set to reverse the direction when the mobile device deviates from it. This allows us to adjust the magnitude of the wind by changing the tilt of the mobile device and gradually increase the magnitude of the direction vector by *α* (see Fig 5).

If you change from Fig 2a and 2b to 2c (or vice versa) of the three angle patterns mentioned above, the direction will be reversed at the moment, causing the problem of sudden popping of simulation or game content. To solve this problem, we blend the angle using the following equation (see Eq 2).

$$\begin{array}{c} v^{*}=v_{2}(\frac{90^{{}^{\circ}}-a_{t}}{10^{{}^{\circ}}})+v_{1}(1-\frac{90^{{}^{\circ}}-a_{t}}{10^{{}^{\circ}}}), \end{array}$$ (2)

where *v*_1_ is the upward direction vector and *v*_2_ is the downward direction vector. Eq 2 is applied only if the angle *a*_*t*_ between the user’s line of sight and the mobile device is 80°≤ *a*_*t*_ ≤ 90°. As a result, when *a*_*t*_ is 80° ≤ *a*_*t*_ ≤ 90°, it is blended and the direction vector is changed smoothly from the upward to the downward. The reference angle 80° we use for blending is a threshold that can be changed by the user. As a result, the direction of the breath is determined by the blended angle, v*.

### Calculating control position with acceleration sensor

In the previous sections, the direction and magnitude of the breath is determined, and in this section the control positions where it is applied are determined. The initial position of the control position can be freely set by the user such as the game character or the sourcing position of the smoke simulation. In this paper, we set initial position of contents as initial position of control position.

**Fig 6:**
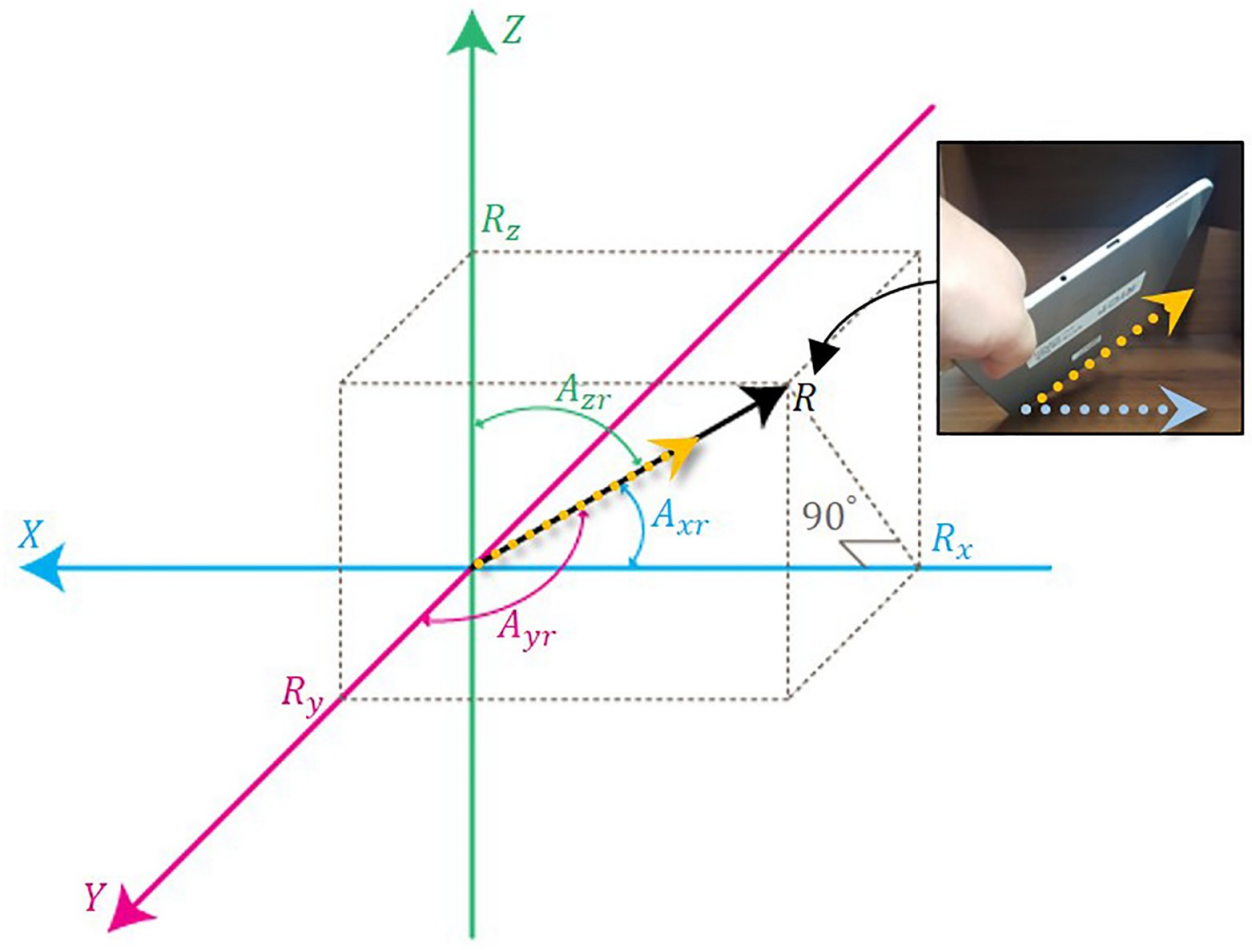
Angle calculation from the axis of the acceleration sensor.

The measured value obtained from the acceleration sensor as the mobile device is tilted is equal to *R* (see Fig 6). *R* is inclined by *A*_*zr*_, *A*_*xr*_, and *A*_*yr*_ from each axis, and the angles for each slope can be calculated using trigonometric functions as Eq 3.

$$\begin{array}{c} cos\left(A_{xr}\right)=\frac{R_{x}}{R},cos\left(A_{yr}\right)=\frac{R_{y}}{R},cos\left(A_{zr}\right)=\frac{R_{z}}{R}, \end{array}$$ (3)

where *R* is the acceleration sensor value measured when the device is tilted. As a result, the angle for each axis can be calculated by Eq 4.

$$\begin{array}{c} A_{xr}=arccos(\frac{R_{x}}{R}),A_{yr}=arccos(\frac{R_{y}}{R}),A_{zr}=arccos(\frac{R_{z}}{R}), \end{array}$$ (4)

We use Eq 4 to calculate the rotation angles for each axis on the mobile device and update the location of the control position according to these calculated angles.

**Fig 7:**
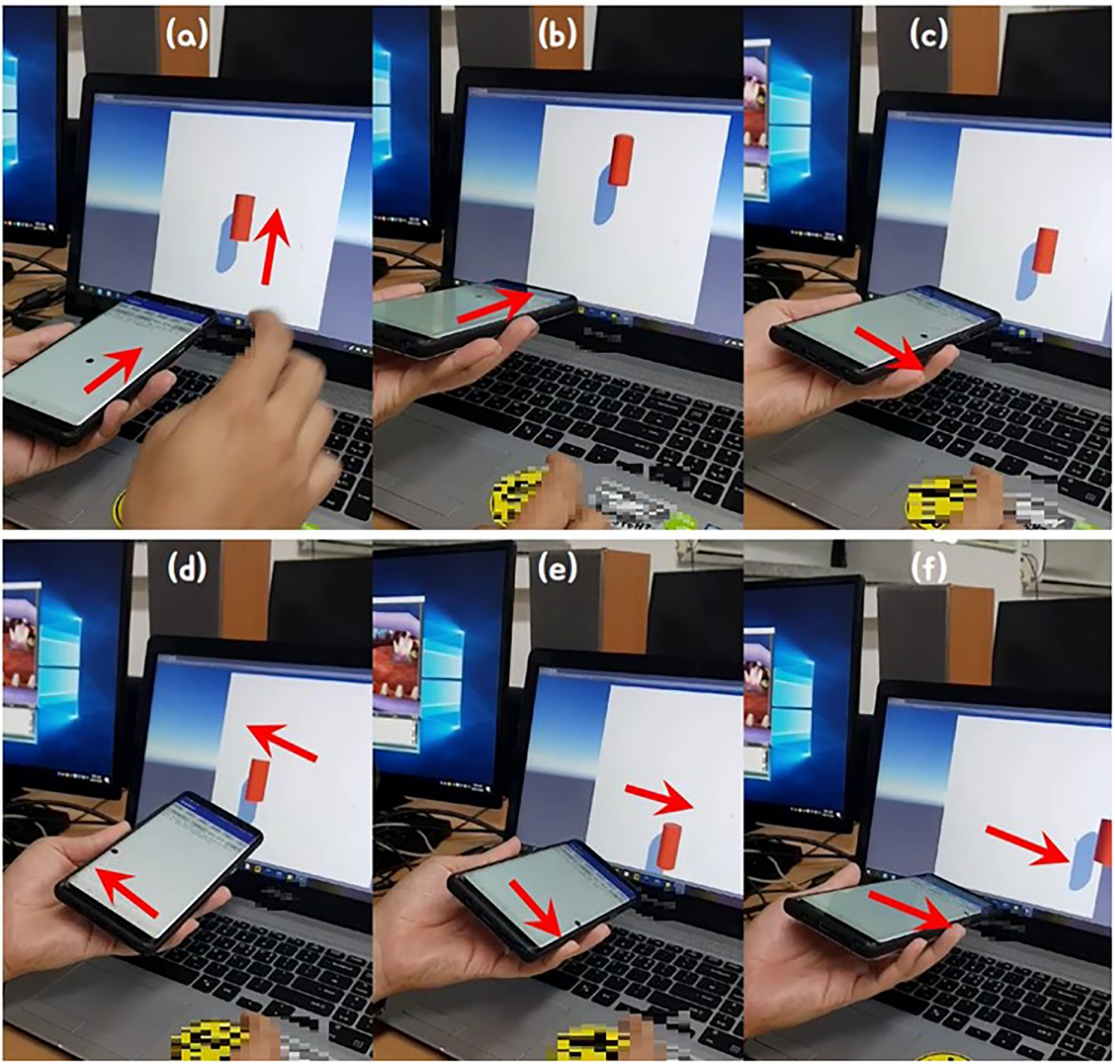
Handling VR content using control position.

Fig 7 shows the red cylinder moving along the tilting direction of the mobile device, which controls the control position just as it controls this cylinder. The more the device is tilted, the faster the cylinder (control point) moves and controls its contents. In order to interact with the control position calculated by the mobile device and the VR content, we used the basic network function provided by Unity3D game engine. Fig 7a is the initial screen. When the breath is blown while the mobile device is tilted, the red cylinder moves in real-time in that direction. Afterwards, when tilting the mobile device upward, the cylinder is also moved upward, and again the device is tilted diagonally downward, so the cylinder moves accordingly (see Fig 7b and 7c). By using this method, we not only control the contents at fixed position, but also freely adjust the target position according to the user’s intention.

### Refining magnitude of breath with sound sensor

**Fig 8:**
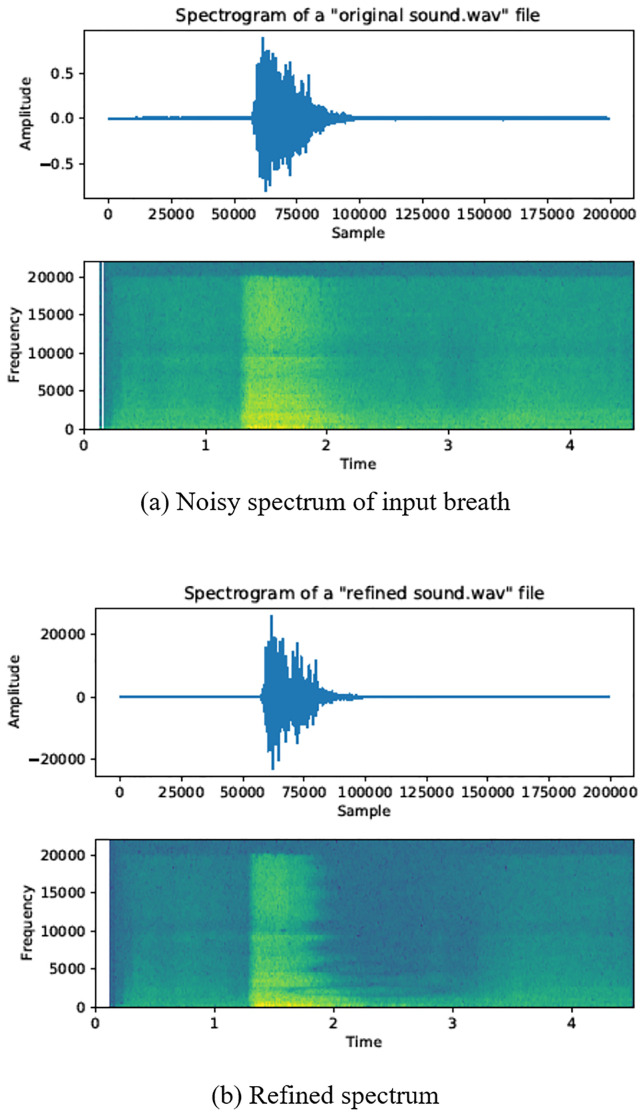
Comparison of breath spectra.

In this paper, we use the sound sensor built into the mobile device to calculate and refine the magnitude of the breath. Most mobile devices use low cost sound sensors and often contain noise. We remove the noise by refining the magnitude of the input sound using a kernel filter that changes over time. The wind that the user actually blows is as shown in Fig 8a. This figure is the spectrum of the sound made by rolling the mouth round. As shown in the figure, the magnitude of the sound increases at the beginning of blowing the breath (see yellow region in Fig 8a), and then rapidly decreases (see green and blue regions in Fig 8a).

**Fig 9:**
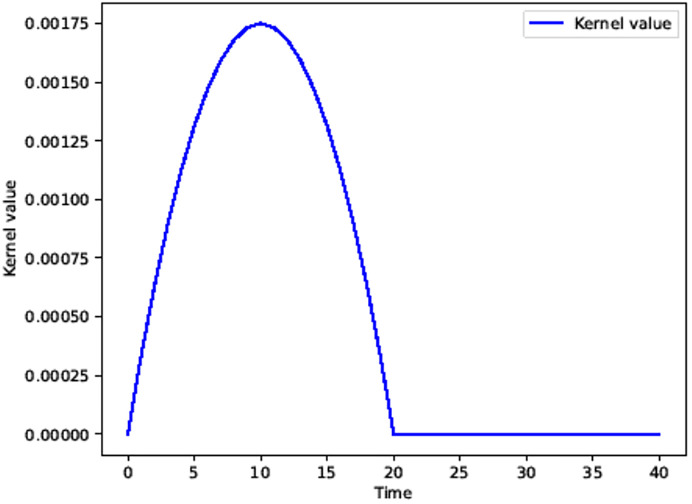
The shape of our breath kernel function inside the support radius *h* = 1.

Low-cost sound sensors on mobile devices are difficult to capture the magnitude of the sound in detail. To compensate for this limitation, we calculate a weighting function to remove the noise contained in the magnitude of the input breath. This function is designed to represent a form similar to the spectrum of a real breath. The detailed method is as follows (see Eq 5).

$$\begin{array}{c} \gamma_{breath}=\frac{0.007}{h^{3.14}}\{\begin{array}{cc} \sqrt{-\frac{4r^{2}}{h}+6r-2h} & 2r>h\;\wedge r\leq h\\ 0 & otherwise \end{array}, \end{array}$$ (5)

where *r* is a value that decreases with time, and in this paper, it is set to gradually decrease from 1 to 0.025, and *h* is a range that affects the magnitude of wind, and is set to 1. Computation of Eq 5 yields a weighting function as shown in Fig 9.

**Fig 10:**
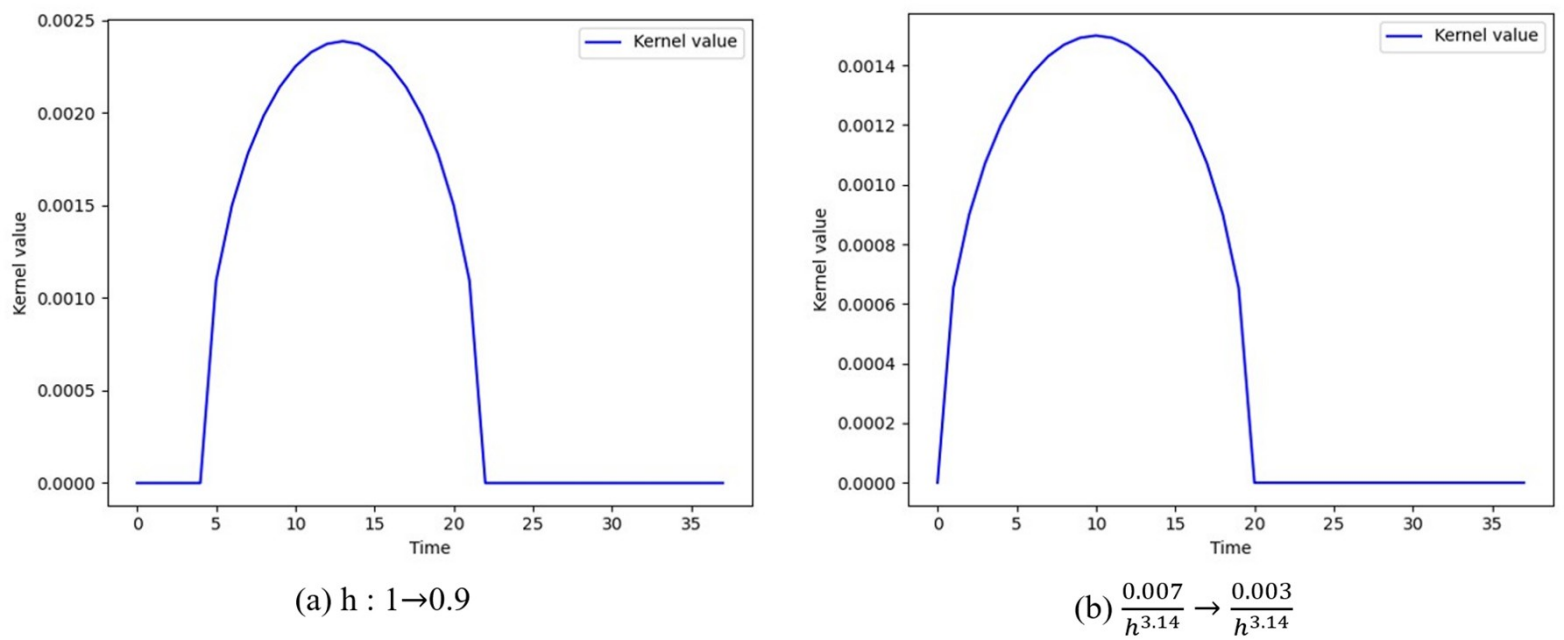
Results with different parameter values.

Fig 10 shows the kernel shape that varies according to the parameter change of the breath kernel function. Fig 10a shows the result according to the change in *h* value. For *h*, the default value of 1 is the maximum value, and if this value is reduced, a delay can be applied at the start of the breath motion. Fig 10a shows the form when *h* is set to 0.9, and delay is applied in the part where the value of the kernel is set to zero, and this value can be adjusted by the user according to the contents. In this paper, we experimented with *h* set to 1. Fig 10b shows a kernel shape that varies according to the weight change of breath kernel. The basic pattern is similar to Fig 9, but when the weight value is small, it can be seen that the size is smaller while maintaining the invert-U shape expressing the patterns of breath.

**Fig 11:**
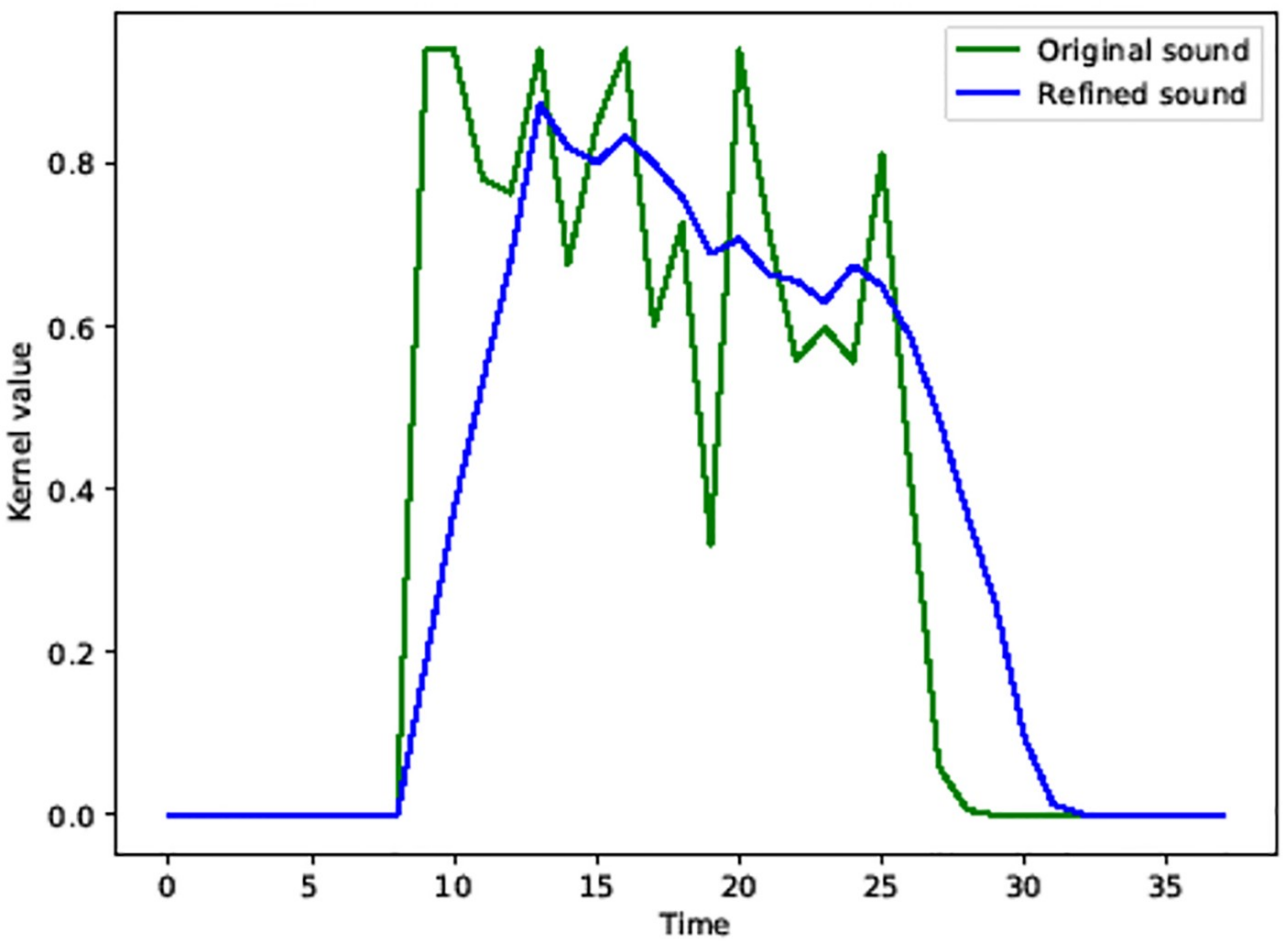
Refined intensity of sound using *γ*_*breath*_ kernel function (green: Original sound, blue: Refined sound).

In this paper, *β*, the final refined magnitude of wind, is obtained by using Eq 6.

$$\begin{array}{c} \beta_{t}=s_{t}\cdot \gamma_{breath}, \end{array}$$ (6)

where *s* is the original magnitude of the breath and *t* is the time. As a result, the magnitude of the breath is greatly affected by gamma during refinement (see Fig 11).

Fig 11 compares the magnitude of the input breath obtained by the sound sensor (green) with the refined magnitude (blue). Compared with the noisy magnitude (green curve) of the input breath from the sound sensor, we can see that the noise is much reduced (blue curve). Comparing the spectra, we can see that the sound features are clearly highlighted (see *X*-axis range 1∼2 in Fig 8b) and the noise is filtered. Comparing the spectrum colors in the area where the magnitude of the sound decreases (see *X*-axis range 2∼4 in Fig 8b), the noise is noticeably removed. In Fig 8a, the area is represented by green mixed with yellow, which contains a lot of noise. On the other hand, in Fig 8b, we can see that the noise is removed to make it clear green.

## Results

### Controlling fluid animations

**Fig 12:**
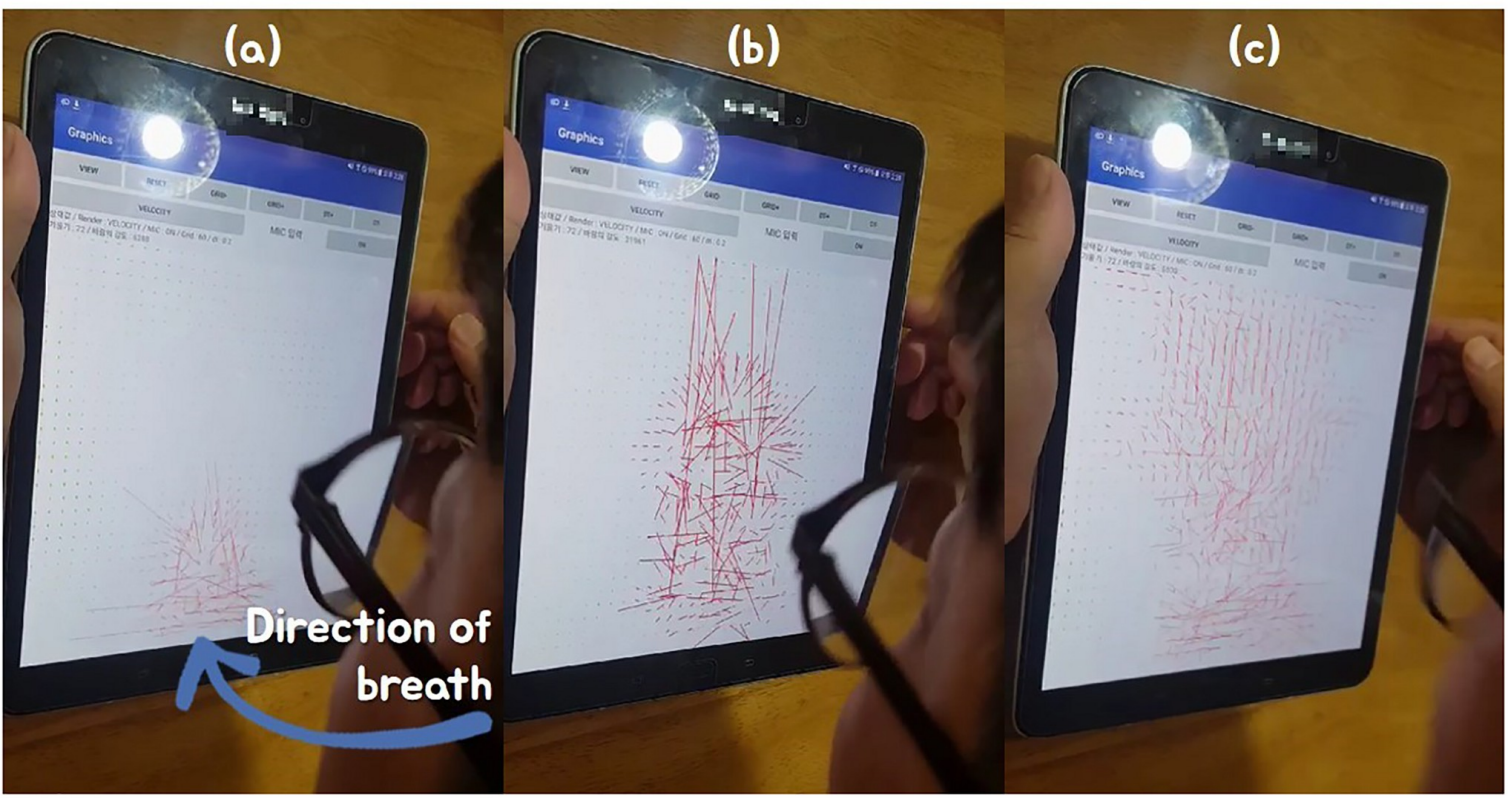
Diffusing vector field by breath.

**Fig 13:**
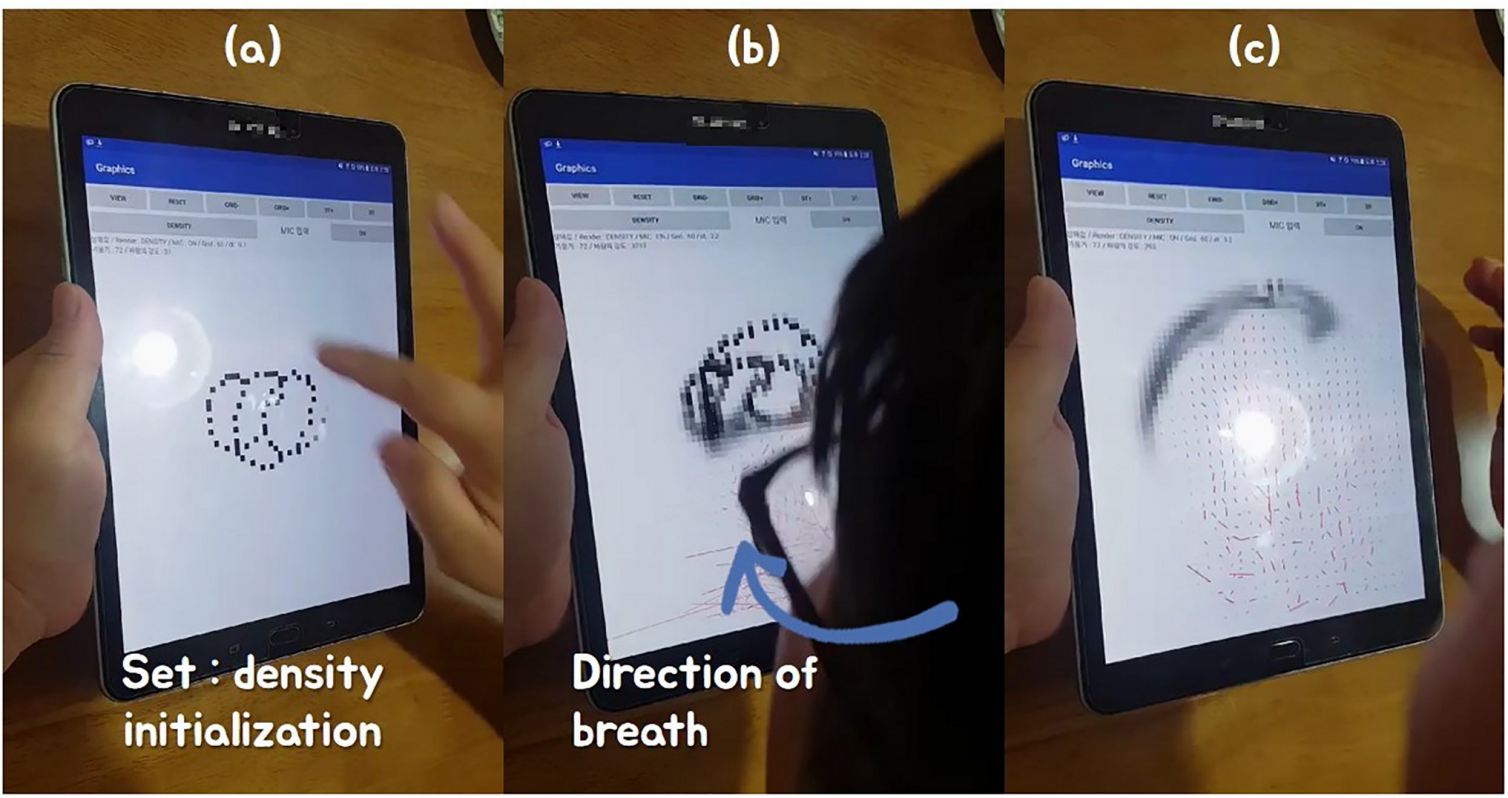
Interactive smoke simulation by breath.

**Fig 14:**
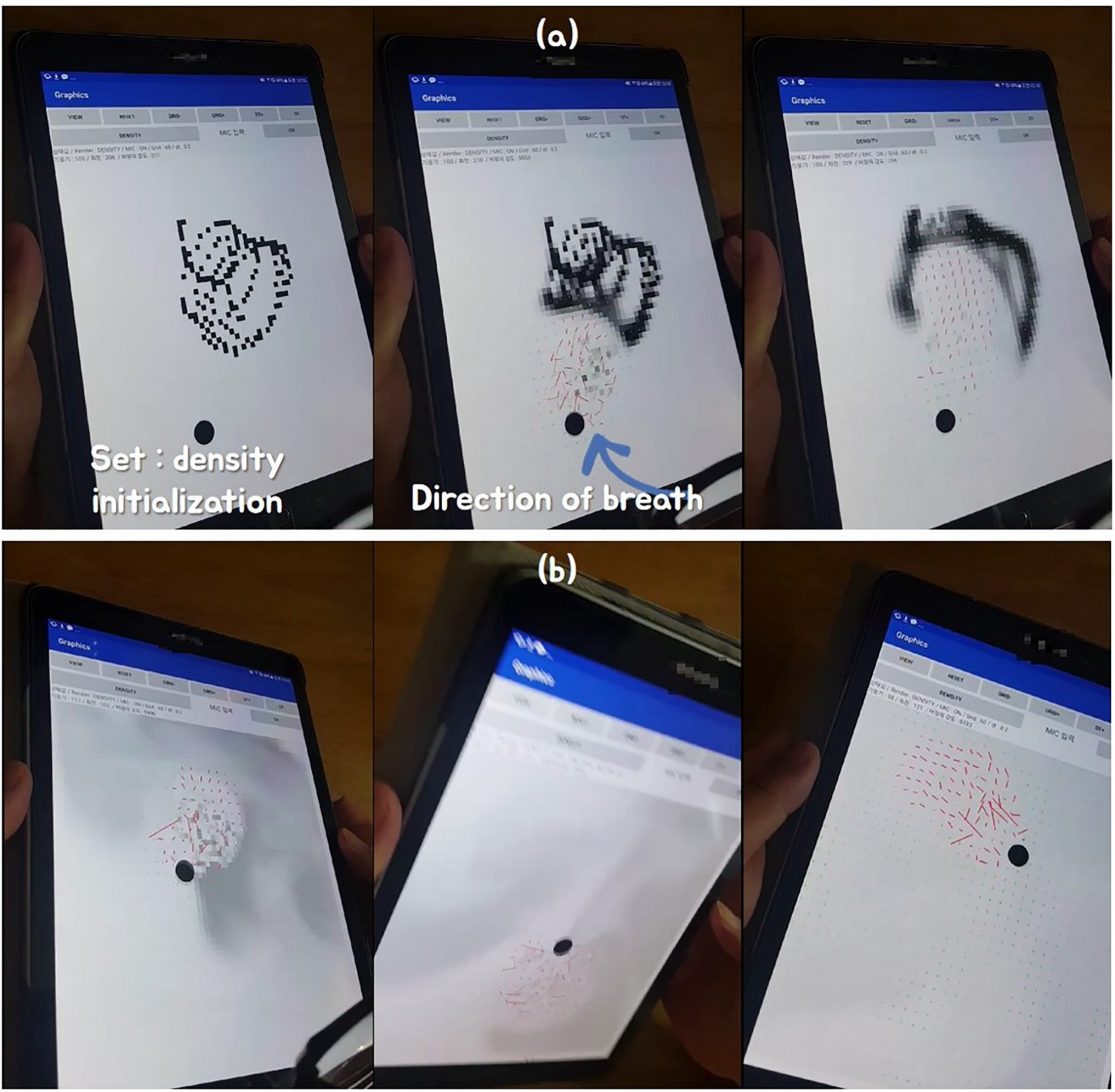
Interaction of user and smoke simulation by control position and breath (black sphere: Control position).

Fig 12 shows the result of the vector field being diffused by the breath. Since the breath is blown with the mobile device facing in front, the result of the vector flowing upward is seen in real-time. Fig 13 shows the result of extending Fig 12 to smoke simulation, and it can be seen that the interaction is real-time according to the direction and size of the breath. In the simulation of Fig 14, the user first enters the initial density value of the smoke, and when the breath is blown, the smoke density is diffused according to the angle pattern between the user and the mobile device.

Fig 14 shows the results of adjusting the control position based on the acceleration sensor and controlling the smoke simulation accordingly. Unlike the result of Fig 13 controlling the smoke using the fixed control position, the user can freely and intuitively control the direction as well as the control position. As a result, smoke simulation reacts in real-time depending on the orientation of the mobile device and the magnitude of the breath (see Fig 14). The smoke simulation solver used in this paper was Jos Stam’s method [44], and the time-step of all smoke simulations was set to 0.2 and their grid resolution was set to 60.

### Controlling games

**Fig 15:**
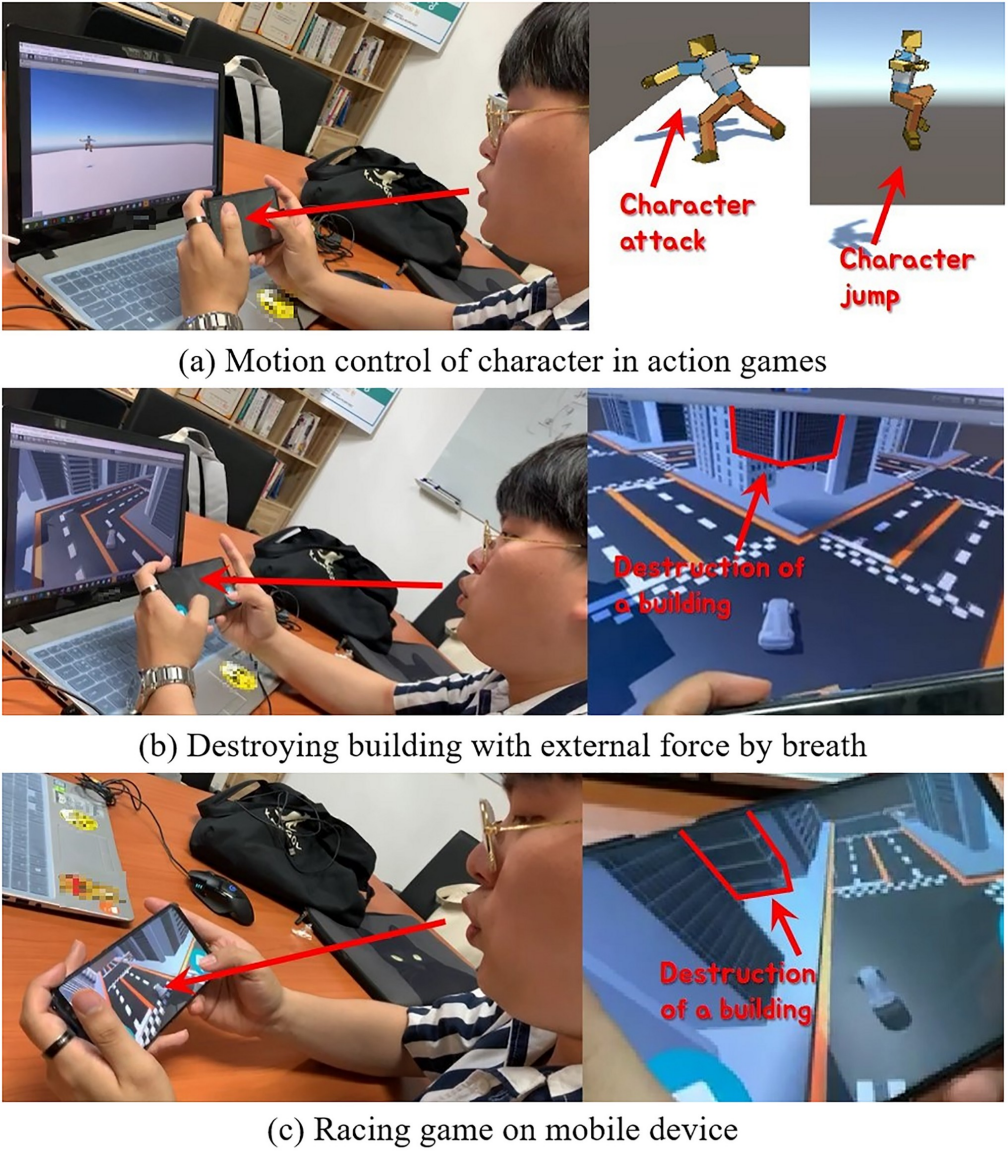
Controlling game objects using breath interface.

Fig 15 shows the results of real-time control of various objects using breath in different kinds of games.

In the first game we are controlling the character’s movement in the fighting game (see Fig 15a). In this result, the breath interface is applied to the character’s attack and jump movements. Basic character movements, except attack and jump, were performed using the gamepad’s navigation keys. By taking actual physical actions on virtual characters in the game, these interfaces can be used to create more interesting contents than the limited interaction of keyboards in environments such as VR and AR.

In the another game, the breath interface was used to destroy buildings by external force in the racing game (see Fig 15b). We tilt the mobile device from side to side to determine the direction in which the racing car will move. Buildings are made to fly in accordance with the breath magnitude and orientation of the user entered into the mobile device. The racing game developed in this paper was provided to 20 experiment participants, and we received feedback from them that it was more fun and immersive than the existing game method controlled by keyboard and mouse (See Section **Evaluation** for more details).

In our study, *γ*_*breath*_ is used to remove noise from the sound sensor, but in the case of sensitivity we additionally found *δ*, which is the minimum magnitude of the breath responding to the mobile device. By excluding sound with magnitude less than *δ* from interaction process, contents could be controlled more stably.

A number of experiments were conducted in various environments to see if there was a delay between the breath interface and the experimental content. As shown in the Result section, there was almost no delay in general situations. However, there is an exception that causes delay when applying the breath interface to the contents. When using a breath interface in a mobile device, we can use the built-in sound sensor, but there is no sound sensor in a general desktop PC. In this paper, a sound sensor of a mobile device is used and breath data obtained from the sound sensor is transferred to a PC through a wireless network. In this process, while transferring data, delay may occur depending on the connection status of the wireless network, but there was little delay in other processes. If it is expanded to multiplayer rather than single player as in this paper, it is considered to be efficient to install the built-in sound sensor on the desktop PC because it is inefficient to transmit breath data of all users to the wireless network.

The proposed technique could control not only fluid simulation but also VR and game contents in real time. Experimental results show that the breath interface can be used to control various kinds of multimedia contents. The parameters in this paper are summarized in Table 1.

**Table 1:** Simulation parameters. We used these specific parameters to generate the example animations shown in this paper.

Name	Description	Value
*α*	Weight for magnitude of the wind direction vector	1.5
*r*	Value that decreases with time	0.025
*h*	Range that affects the magnitude of wind	1.0

## Evaluation

Preliminary user study was conducted to understand the effect of the proposed breath interface on controlling various contents. A total of 20 participants (10 normal and 10 disabled people) were tested with action and racing games using the breath interface. Participants in the validation test are 10 college students with the following disabilities: 5 people with hand discomfort, 3 people with wheelchair, 2 people with uncomfortable upper body movement.

Both games for applying the proposed framework were developed by ourselves, and the operation method is as follows. First, in the action game, a basic motion that can control the movement of the character is assigned to the keyboard, and the type of character is one in the game. The basic movement of a character can be moved using “left, right, forward, and backward”, and it has been developed to perform movements such as jump and attack with special keys. We experimented by connecting the special key to the breath interface. In this process, the terrain was set in a simple flat type, and NPCs were not added separately. In this experiment, we observed whether jump or attack motion is naturally operated when the user suddenly takes a breath motion while moving the character freely using the keyboard. In the racing game, the keyboard was used to move the car directly, and the special key was set to perform an attack. Here, the attack destroys the building that exists in front of the direction in which the car moves, and it is connected to the breath interface. Since the breath interface can only express the on-off of the attack, the building that is in the front direction of the car and within the radius defined by the user was destroyed. In this game, the city terrain was automatically synthesized based on the current position of the car, and collision processing between the car and the building was not applied. Also, we experimented with this game on mobile devices. Both games mentioned above used sound sensors provided by mobile devices, and were developed to deliver breath data in real time through the network provided by Unity3D.

### User study design

After a brief description of how to play the game, participants use the breath interface to play action and racing games. Each game was run in random order, giving each game about 10 minutes of play time. Each game was played twice before and after applying the breath interface.

In the action game, participants were asked to complete the game by killing all non-player characters (NPCs) randomly placed on the terrain. Before the game, participants were free to choose which actions (attack, jump, and run) to apply to the breath interface. There was no change in the difficulty of the game, and the number of terrains and NPCs were adjusted randomly each time. There was no time limit for the game, and killing all NPCs made the game over.

In a racing game, we randomly placed buildings or roads and asked participants to play the game until they reached the destination. Unlike the action game, the breath interface is limited to being used only to blow up buildings. There was also no time limit for the game.

### Summarization

Participants were asked to complete the following questionnaire after playing the game before and after applying the breath interface to each game (see Table 2). Basically, we examined how the breath interface affects the fun and operability of the game. In addition to immersion, we conducted a survey by dividing the normal and the disabled to see how the disabled evaluate our proposed interface.

**Table 2:** Questionnaire (Q1,Q2,Q4: Satisfied(5), Average(3), Not Satisfied(0), Q3: Easy(5), Normal(3), Difficult(0)).

Q1	Was the fighting game with the breath interface more fun and interesting than using the gamepad alone?
Q2	Was the racing game with the breath interface more fun and interesting than using a gamepad alone?
Q3	Was it difficult to operate the breath interface?
Q4	(Only handicapped student) Do you think the breath interface has helped you in using the content?

**Fig 16:**
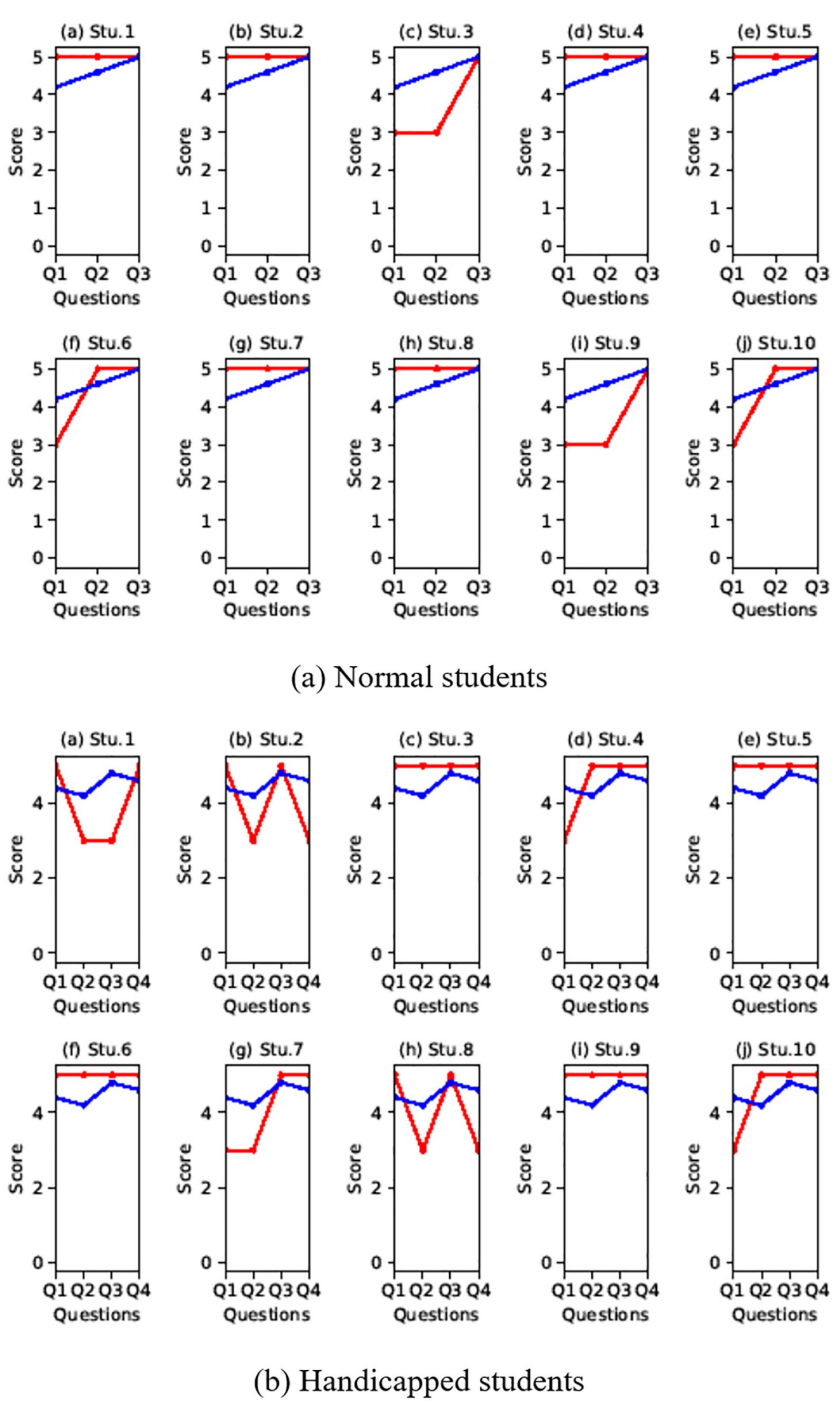
Survey students (normal studuent: 10, handicapped student: 10, red line: Score for the question, blue line: Avg. score for the question).

Fig 16a shows the results of a survey of participants without disabilities. The average score was 4.5 for all questions, and most participants were satisfied with the breath interface. Looking at the comments from the participants, many thought that the expectations before the experiment were not high because they did not know how the breath would be applied to the game. Nevertheless, many commented that it was interesting to introduce new user gestures away from the way content was previously operated only with gamepads. These positive ratings are well illustrated in Fig 16a, especially in the racing game rather than the action game. The reason for this result is that action such as attack or jump is considered to be less immersive because these actions are not intuitively consistent with breath. On the other hand, the action of blowing buildings through the breath in the racing game seems to be more interesting or immersive because there is an intuitive correspondence with the breath.

Fig 16b is the result of a questionnaire from people with disabilities, and we received a positive evaluation from most of them. Q4 was given only to people with disabilities and is a question to investigate how positively the proposed interface has affected not only the game but also one’s disability. For this question we mostly got a good score. There were some individual differences in this question. In particular, low scores were often assessed when the breath interface was not associated with their disability (see Stu.2 and Stu.8 in Fig 16b). Among the participants, wheelchair users gave a relatively low score. In fact, they seemed to be more comfortable to use their fingers than to the breath interface, since only the movements of the lower body are uncomfortable and the movements of the upper body are not different from the general public. Nevertheless, most participants were positive about factors such as game play and fun. In addition, there were some comments that it was sometimes difficult to blow the breath continuously.

In this paper, two demo games produced for validation test were not set to allow users to precisely control attacks, and there was no difference between the general public and the disabled. However, each user chose either attack or jump to apply to the character (see Fig 15a). Since the complicated continuous motion is difficult to control with the breath interface, it was applied to a simple action game. In the racing game, a user can select the movement to apply to the game, just like the action game. It can be used as an external force to blow up surrounding objects, as shown in Fig 15a, and otherwise it can be used to accelerate cars. When used as a means to accelerate, the degree of acceleration is controlled by the intensity of the wind, and the direction can be changed by controlling the direction with a finger. However, most participants used the breath interface as an external force. The reason was that when the wind blew, it was more intuitive and fun to fly buildings than to accelerate the vehicle.

**Fig 17:**
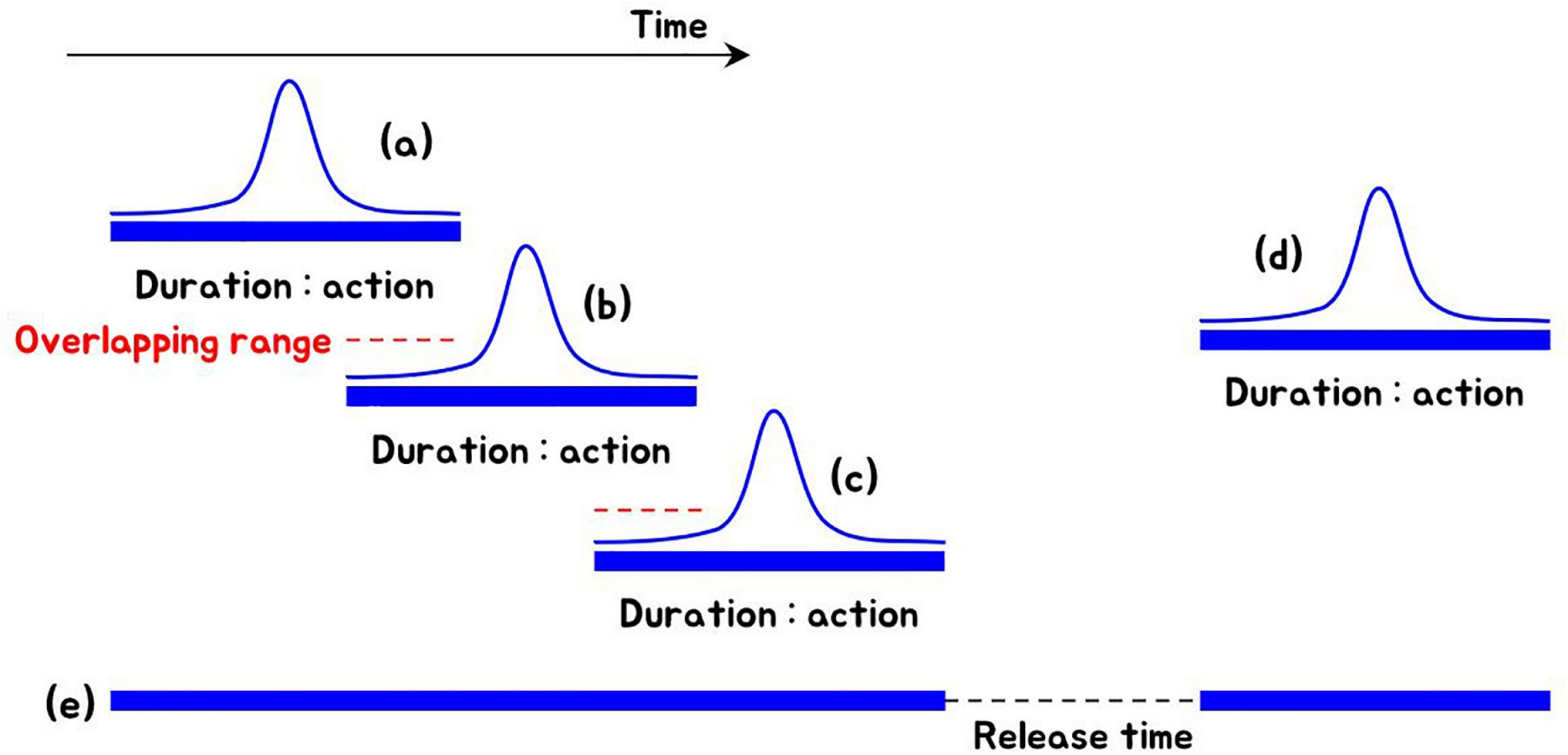
Operation method for controlling objects in racing and action games.

When the breath interface was applied to action and racing games, the game object was consistently controlled even if the wind was repeatedly blown within a short period of time. As mentioned before, the breath generally appears in a pattern similar to the Gaussian kernel (see Fig 9), and when the wind blows several times within a short period of time, it is expressed as the pattern shown in Fig 17a–17c. Multiple winds in a short period of time will cause overlapping areas (see red dotted lines in Fig 17), causing the contents control to become unstable. We handled it with the On-Off flag regardless of the intensity of the wind. So, even if the sound is overlapped and loud, the state remains On, so there is no problem (see Fig 17e). When the user does not blow the wind, it is switched to Off (see release time in Fig 17), and when it is blown again, it is switched to On, so the game can proceed naturally (see Fig 17d). This method is used when applied in the game, and is applied differently in the case of fluid animation. In fluid animation, since the breath is added as the external force of the Navier-Stokes equation, when the wind blows repeatedly within a short period of time, the increased intensity of the wind is transmitted to the external force completely. The methods described above are examples of how to combine the proposed breath interface with various content, so how to influence the content can be selected and developed in the way the developer wants (On-Off mode or the magnitude mode).

In this study, training time of about 10 minutes was given for each game, and the game was played twice before and after applying the breath interface. Because the breath interface operates intuitively, it was not very difficult to learn or play the game. In fact, we tried increasing the training time or the number of times, but there was little difference in the user’s skill level, and in about 10 minutes, anyone could easily participate in the experiment. We experimented with 10 normal students and 10 handicapped students each. Participants were 20-23 years old, and the normal students consisted of 6 males and 4 females, and the handicapped students consisted of 7 males and 3 females. We announced that we will be experimenting with the game with a new interface before gathering the participants, and most of the participants have experience with game manipulation and enjoy the game. Handicapped students suffered from lower body disorders (7 people), Down syndrome (1 person), and hearing impairments (2 people). Of course, the evaluation of interfaces differed according to the types of disabilities. Among the normal students, there were opinions that it was interesting to control NPCs using breath, not just fingers, and among handicapped students, students with lower body disorders or hearing impairments showed a lot of interest. Students with disabilities with limited movement showed interest in being able to control distant contents with breath instead of fingers. In addition, there was an opinion that future development is expected in that interaction can be possible without being spatially close. There was an opinion that as much as using breath motion, connecting with VR contents that sings or plays musical instruments will improve immersion, and it will be helpful for learning to play itself.

In addition, we analyzed the reliability coefficient of the survey conducted earlier through *Cronbach*′ *s* *alpha* technique (see Tables 3 and 4). Analysis of the experimental data with normal and handicapped students was conducted, and *Cronbach*′ *s* *alpha* value was not large (see Tables 3 and 4). The reason why the analysis results are like this is because the number of data samples is too small rather than there is a problem in the questionnaire, so it is not likely that reliable results have been produced.

**Table 3:** Cronbach’s alpha with normal student data.

Normal student	Q1, Q2, Q3	Sum of Q1∼3
*Stu*.1	5, 5, 5	15
*Stu*.2	5, 5, 5	15
*Stu*.3	3, 3, 5	11
*Stu*.4	5, 5, 5	15
*Stu*.5	5, 5, 5	15
*Stu*.6	3, 5, 5	13
*Stu*.7	5, 5, 5	15
*Stu*.8	5, 5, 5	15
*Stu*.9	3, 3, 5	11
*Stu*.10	3, 5, 5	13
Variance	1.066, 0.711, 5	2.844
Variance sum	1.777	–
*Cronbach*′ *s* *alpha*	0.563	–

**Table 4:** Cronbach’s alpha with handicapped student data.

Handicapped student	Q1, Q2, Q3, Q4	Sum of Q1∼4
*Stu*.1	5, 3, 3, 5	16
*Stu*.2	5, 3, 5, 3	16
*Stu*.3	5, 5, 5, 5	20
*Stu*.4	3, 5, 5, 5	18
*Stu*.5	5, 5, 5, 5	20
*Stu*.6	5, 5, 5, 5	20
*Stu*.7	3, 3, 5, 5	16
*Stu*.8	5, 3, 5, 3	16
*Stu*.9	5, 5, 5, 5	20
*Stu*.10	3, 5, 5, 5	18
Variance	0.933, 1.066, 0.4, 0.711	3.555
Variance sum	3.111	–
*Cronbach*′ *s* *alpha*	0.166	–

## Discussion and implementation

**Fig 18:**
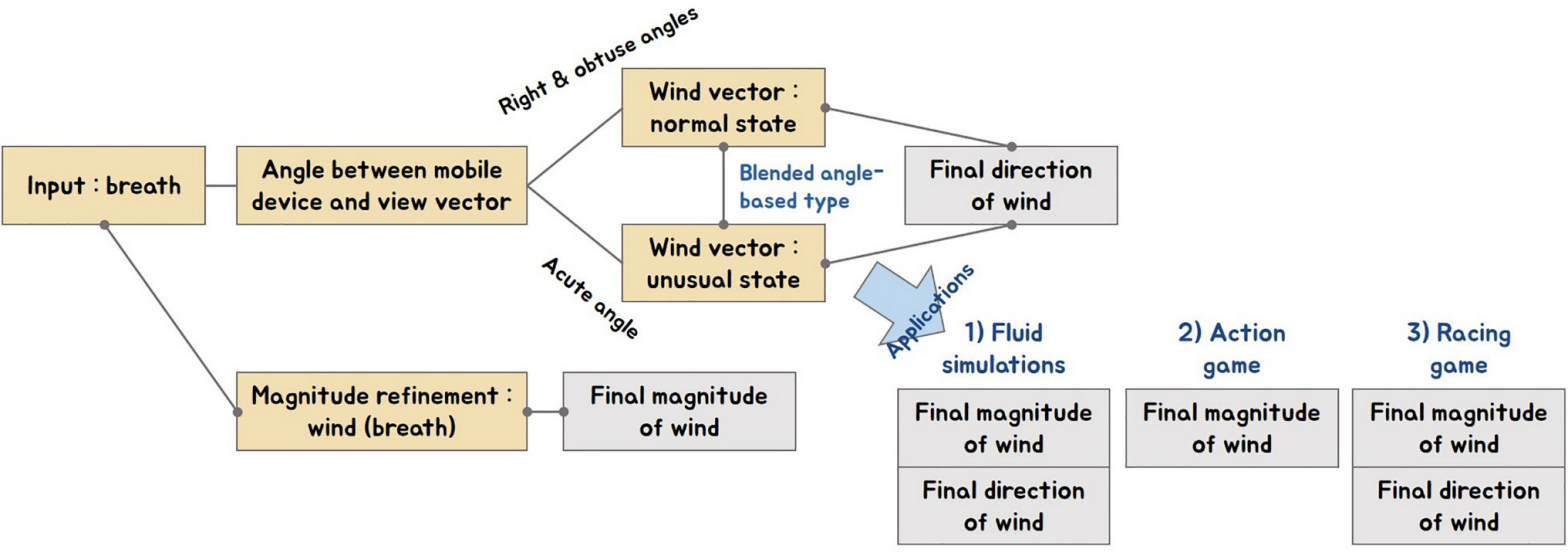
Activity diagram with our method.

In this section, how to use the framework proposed in this paper for fluid simulations and games, etc. is explained from the perspective of implementation. Fig 18 is an activity diagram, the upper part shows the algorithm overview of the breath interface, and the lower part explains the method used at the application level. When the user blows the input breath on the mobile device, 1) the direction of the wind is determined by using the angle between the mobile device and the user’s view vector. In this process, the blended angle method is used to model the direction to change naturally. 2) When breath data is input to the sound sensor built into the mobile device, the magnitude of the wind is refined by the *γ*_*breath*_ kernel, resulting in a smoothed magnitude. The breath interface was applied to fluid simulations, action and racing games by using the obtained direction and magnitude of wind, and the wind properties described earlier can be applied according to the characteristics of the content. In fluid simulations, external force was applied to the smoke density by using the direction and magnitude of the wind, and in games, the movement of NPC was controlled using only the breath’s on-off flag.

As mentioned in the related work section, the recent flow of the VR interface is not a simple input such as a mouse click or a screen touch, but reflects the physical environment as much as possible and attempts to communicate with the NPC or application by performing actions similar to the real one. The technique proposed in this paper is also an interface that provides intuitive interaction through the user’s breath data. There have been steady studies to reduce the heterogeneity of physical interface when users interact with contents in virtual environment: 1) A study to make an interaction with a device similar to a real chopstick shape to reduce the heterogeneity of chopstick motion, 2) A device that can handle the action of catching a gun in the game and the ready posture for shooting. These studies aim to increase the “interaction accuracy” by considering the physical environment as much as possible when users use contents. However, this study is a concept such as “interaction helper” to give additional effects such as fun, immersion, and interest to users while maintaining only the aspect of using interface like the existing study. For example, if the behavior of the NPC blowing the wind can be controlled through the user’s breath, the immersion will also be improved for users of the content. However, this does not mean to disable the existing interface using hands and feet, but to obtain synergy effects by using it with the proposed method. The proposed technique can be used for all contents that want to utilize a breath-based interface, such as blowing out candles in a VR environment or creating a VR artwork that blows sand or spreads paint through the breath. There have been studies using breath data in previous studies, but most of them have focused on improving the accuracy of recognition using breath biometrics. Also, there is a lot of noise involved in building the interface using raw breath data, so it is not only less accurate, it is not enough to control the contents. We have alleviated this problem and introduced a robust breath interface, and experimented by applying it to physically-based simulations and various game applications. The proposed interface also received positive reviews from users with certain disabilities. In the future, if you use functions frequently used for general data editing, such as video playback, picture file control, and file saving/opening, as breath input, it will be an interface that is useful for people with disabilities and those who have difficulty recognizing or controlling objects.

### Implementation: Coupling with Navier-Stokes equations

Now that we have demonstrated the ability of novel interface to adjust position of contents with interesting external force based on breath, in this section we couple the breath force to the two-dimensional Navier-Stokes equations. In order for the breath interface to be converted into a vector field, the equation must be re-formed considering the breath in the Navier-Stokes equation, the governing equation of the fluid. To calculate the differential form of the momentum equation representing fluid motion, we apply Newton’s Second Law for fluid particles having mass dm as follows. Newton’s second law on the finite system is as follows (see Eq 7):

$$\begin{array}{c} F=\frac{P}{dt}, \end{array}$$ (7)

where linear momentum *P* is calculated by the following equation (see Eq 8).

$$\begin{array}{c} P=\int_{mass}udm, \end{array}$$ (8)

Newton’s second law on mass *dm* in a continuum system can be written as follows (see Eq 9):

$$\begin{array}{c} dF=dm\frac{du}{dt}, \end{array}$$ (9)

After obtaining the acceleration equation for the particle of mass *dm* moving in the velocity field, Newton’s second law can be rewritten as a vector equation as follows (see Eq 10):

$$\begin{array}{c} dm=\frac{Du}{Dt}=dm(\frac{\partial u}{\partial t}+u\cdot u)=dF, \end{array}$$ (10)

Here, it is important to consider the force *dF* acting on the differential elements of the mass *dm* and volume *dV* = *dxdydz*. In general, in fluid dynamics, *dF* is composed of surface force and body force as shown in the following equation (see Eq 11).

$$\begin{array}{c} dF=dF_{surface}+dF_{body}, \end{array}$$ (11)

The surface force can be rewritten using viscosity force and pressure gradient as shown below (see Eq 12).

$$\begin{array}{c} dF_{surface}=\{\mu \nabla \cdot (\nabla u)-\nabla p\}dV, \end{array}$$ (12)

Body force is the force exerted on the entire finite element, and in the general Navier-Stokes equation, gravity is the only body force acting on the fluid. Instead of gravity, the body force can be rewritten using the breath-based external force described above (see Eq 13).

$$\begin{array}{c} dF_{body}=U_{potential}dV, \end{array}$$ (13)

Finally, the body force is calculated with gravity as shown below(see Eq 14).

$$\begin{array}{c} U_{potential}=U_{gravity}+U_{buoyancy}, \end{array}$$ (14)

where *U*_*wind*_ is the external force generated by the breath, and the Navier-Stokes equation is re-formed as follows (see Eq 15):

$$\begin{array}{c} \frac{\partial (\rho u)}{\partial t}+\nabla \cdot (\rho uu)=\mu \nabla \cdot (\nabla u)-\nabla P+\rho g+U_{wind}, \end{array}$$ (15)

The only difference between Eq 15 and the general Navier-Stokes Equation is the presence of *U*_*wind*_, a potential force defined by the user. *U*_*wind*_ is easily combined with the existing fluid equation, because it is calculated only when the user blows, there is not much additional calculation.

## Conclusions and future work

In this paper, we propose a new breath interface framework using an angle blending technique and an easy way for the user to control the target position to handle contents. We show the effectiveness of the proposed method by using it for fluid animations and game contents control. When controlling the control position, the acceleration sensor built into the mobile device was used so that the user can freely adjust the position where the interaction is to be applied. As a result, the user can freely change the control position by tilting the device. By calculating the direction of the breath using the angle blending method, we solved the problem of suddenly changing the direction of the breath when rotating the mobile device. By applying the proposed technique to fluid animation, VR, and game contents, we can create an application that can be freely controlled in real-time by breath.

The proposed framework can be used not only for fields requiring large movements but also for places where more detailed interaction is required (games, arts, etc.). In addition, it can be used for healthcare, welfare technology, etc. for those who are not comfortable or agile, such as children, the disabled and the elderly. Since the proposed system does not fully include the VR environment, there are some problems to be solved in order to integrate it into various VR contents. Since most VR contents are based on HMD, it is difficult to intuitively integrate the breath interface. To solve this problem, new devices and interfaces that can be combined with the HMD will be required. Another problem arises from noise mixed with the breath. It is very difficult to completely remove the noise from the breath coming from the microphone sensor. In this paper, we calculated the intensity of the breath by minimizing the noise by using a kernel similar to the actual pattern, but it may be different from the actual one. In addition, it is more difficult than using the keypad method if you repeatedly blow the breath frequently. Therefore, in order to apply to various environments, it is necessary to improve the method to prevent frequent breath blowing, or to expand it to a way that can control contents more easily through the breath. We plan to study the breath interface for the elderly and people with disabilities to work in a cloud computing environment.

## Supporting information

S1 Video Supplementary result data. Related to Figs 12–15.
(AVI)
